# Involvement of Type 10 17β-Hydroxysteroid Dehydrogenase in the Pathogenesis of Infantile Neurodegeneration and Alzheimer’s Disease

**DOI:** 10.3390/ijms242417604

**Published:** 2023-12-18

**Authors:** Xue-Ying He, Jannusz Frackowiak, Carl Dobkin, William Ted Brown, Song-Yu Yang

**Affiliations:** 1 Department of Molecular Biology, NYS Institute for Basic Research in Developmental Disabilities, Staten Island, NY 10314, USA; 2 Department of Developmental Neurobiology, NYS Institute for Basic Research in Developmental Disabilities, Staten Island, NY 10314, USA; 3 Department of Human Genetics, NYS Institute for Basic Research in Developmental Disabilities, Staten Island, NY 10314, USA; 4 Ph.D. Program in Biology-Neuroscience, Graduate Center of the City, University of New York, New York, NY 10016, USA

**Keywords:** ABAD, Alzheimer’s disease, 17β-HSD10, mitochondria, multifunctional protein, neurosteroid metabolism

## Abstract

Type 10 17β-hydroxysteroid dehydrogenase (17β-HSD10) is the *HSD17B10* gene product playing an appreciable role in cognitive functions. It is the main hub of exercise-upregulated mitochondrial proteins and is involved in a variety of metabolic pathways including neurosteroid metabolism to regulate allopregnanolone homeostasis. Deacetylation of 17β-HSD10 by sirtuins helps regulate its catalytic activities. 17β-HSD10 may also play a critical role in the control of mitochondrial structure, morphology and dynamics by acting as a member of the Parkin/PINK1 pathway, and by binding to cyclophilin D to open mitochondrial permeability pore. 17β-HSD10 also serves as a component of RNase P necessary for mitochondrial tRNA maturation. This dehydrogenase can bind with the Aβ peptide thereby enhancing neurotoxicity to brain cells. Even in the absence of Aβ, its quantitative and qualitative variations can result in neurodegeneration. Since elevated levels of 17β-HSD10 were found in brain cells of Alzheimer’s disease (AD) patients and mouse AD models, it is considered to be a key factor in AD pathogenesis. Since data underlying Aβ-binding-alcohol dehydrogenase (ABAD) were not secured from reported experiments, ABAD appears to be a fabricated alternative term for the *HSD17B10* gene product. Results of this study would encourage researchers to solve the question why elevated levels of 17β-HSD10 are present in brains of AD patients and mouse AD models. Searching specific inhibitors of 17β-HSD10 may find candidates to reduce senile neurodegeneration and open new approaches for the treatment of AD.

## 1. Introduction

**Figure 1:**
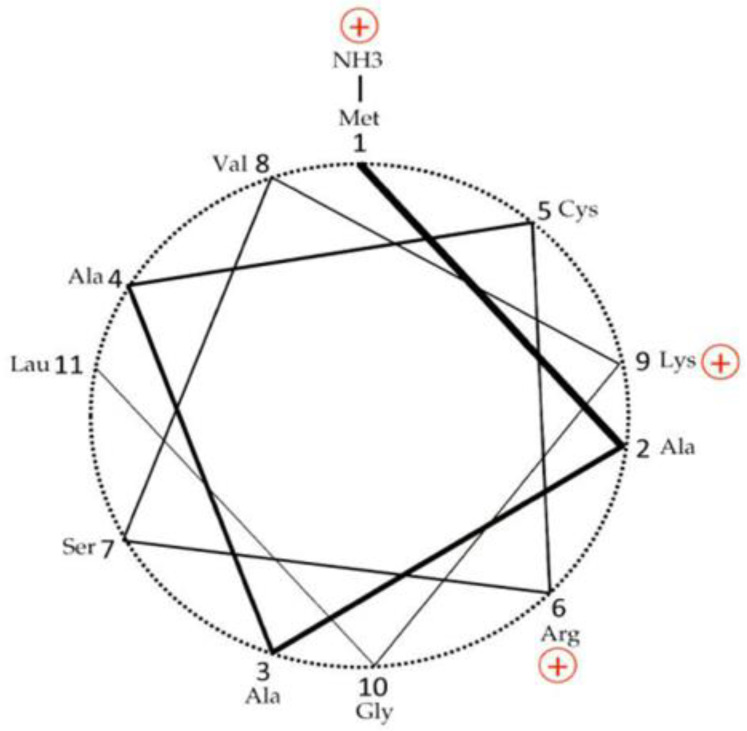
N-terminal mitochondrial targeting sequence of 17β-HSD10 from human [1,2,3] and rat [32] displayed as ‘helical wheel’ (3.6 residues per turn), showing an amphiphilic helix. Adapted from Figure 5 of Ref. [31].

The *HSD17B10* gene (Gene ID: 3028—*HSD17B10*) was first cloned from human brain and mapped to Xp11.2 in 1997 by He et al. [1]. Its product, type 10 17β-hydroxysteroid dehydrogenase (17β-HSD10) (OMIM 300256—17beta-hydroxysteroid dehydrogenase X.) is a mitochondrial, homo-tetrameric protein composed of 1044 amino acid residues with a molecular weight of 108 kDa, also known as human brain short-chain L-3-hydroxyacyl-CoA dehydrogenase (SCHAD) [1,2,3,4,5] as a member of the short-chain dehydrogenase/reductase family [1,2,3,4,5,6] or 3-hydroxy-2-methylbutyryl-CoA dehydrogenase [7,8,9,10,11,12,13,14,15,16]. 17β-HSD10 is the main hub of exercise-upregulated mitochondrial protein [17]. It had also been named as the endoplasmic reticulum-associated Aβ-binding protein (ERAB) [18,19,20,21,22] and renamed again as Aβ-binding alcohol dehydrogenase (ABAD) based upon so-called *generalized* alcohol dehydrogenase activities (C2-C10) [21,22,23]. The ERAB/ABAD was well known for the mediating of Aβ neurotoxicity to destroy brain cells (see Figure 1 of Ref. [18]). ABAD was first reportedly associated with the endoplasmic reticulum same as the ERAB [18,19,20,21,22] because it reportedly had an ‘ER targeting signal’ [20], although human L-3-hydroxyacyl-CoA dehydrogenase type II (HADH2), the equivalent of ERAB/ABAD [21,22] was never isolated from microsomes the endoplasmic reticulum [18,19,20,21,22,23,24,25,26,27,28,29,30]. After a mitochondrial targeting signal was found in the N-terminal of 17β-HSD10/SCHAD [2,3,31] (see Figure 1), it was followed by a claim [23] that ABAD was a mitochondrial protein without 17β-HSD10/SCHAD literature being cited.

A mystery emerged—why human ABAD had beenrecognized as an equivalent to ERAB [21,22,23,24], a peptide with molecular weight 27 kDa only [18,19,20,21,22] even if rat ABAD was reportedly a tetramer later [30] same as rat 17β-HSD10 [32]. Unfortunately, there have been no corrigenda of ERAB or ABAD available in the prestigious journals *JBC* or *Nature* to date, although human 17β-HSD10, a multifunctional protein [33], was well recognized to be important to the pathogenesis of neurodegeneration [1,2,3,33,34,35,36].

## 2. Dehydration of Straight or Branched Chain Acyl-CoA Derivatives

**Figure 2:**
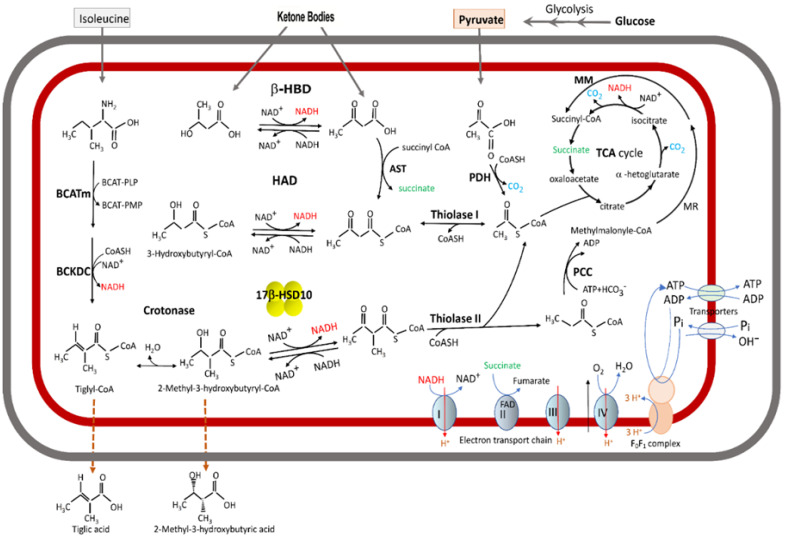
Acyl thioester metabolism and oxidative phosphorylation in brain mitochondria. The fatty acid β-oxidation pathway is shown only the second half here. A cluster of four yellow balls represents 17β-HSD10/HADII. β-HBD rather than 17β-HSD10/HADII or HAD plays a key role in the ketone body metabolism. A missense mutation at 17β-HSD10/HADII would block the isoleucine catabolic pathway to result in the accumulation of isoleucine metabolites so that glucoronated tiglic acid and 2-methyl-3-hydroxybutyric acid are excreted from HSD10 deficiency patients’ urine. Adapted from Figure 1 of Ref. [47].

The first catalytic function of the *HSD17B10* gene product 17β-HSD10 was found to be its L-3-hydroxyacyl-CoA dehydrogenase (HAD) activity [1]. Since it can catalyze the β-oxidation of branched-chain fatty acyl-CoAs (see Figure 2), 17β-HSD10 belongs to the HADII family so it has an alternative name as SDR5C1 [37,38,39]. In contrast, L-3-Hydroxyacyl-CoA (HADH) dehydrogenase or medium-chain/short-chain HAD (EC 1.1.1.35) is a dimer with a molecular weight of 66 kDa. All HADHs including the membrane-associated long-chain HADH catalyze the third step of the fatty acid β-oxidation [40,41,42,43] (see Figure 1 of Ref. [43]) but have no role to play in the β-oxidation of the branched-chain fatty acyl-CoA derivatives [44,45] (see Figure 2). On the other hand, HADH deficiency known by clinicians as short-chain 3-hydroxyacyl-CoA dehydrogenase (SCHAD) deficiency (OMIM#231530) is a fatty acid oxidation disorder causing hyperinsulinism in pediatric patients [46], unrelated to 17β-HSD10 indeed even if both could catalyze the oxidation of acyl-CoA derivatives in mitochondria to provide energy.

The HADII, a new kind of HAD, was first isolated from rat [7] and bovine sources [39]. Such HADII enzymes catalyze the oxidation of 2-methyl-3-hydroxyacyl-CoA. They belong to the HADII/SCHAD family [34], being homologs of human type 10 17β-hydroxysteroid dehydrogenase [38,45] (see Figure 2). The human brain SCHAD/17β-HSD10 [1] was later found to be a multifunctional protein [2,3,5,6,7,8,9,10,30,44,45], the same as other members of the SDR family [6,33,34,44], and therefore it was redesignated according to the international nomenclature system as type 10 17β-hydroxysteroid dehydrogenase [3,47,48,49].

## 3. 17β-HSD10 as a Multitask Enzyme Involved in Different Metabolic Pathways

**Figure 3:**
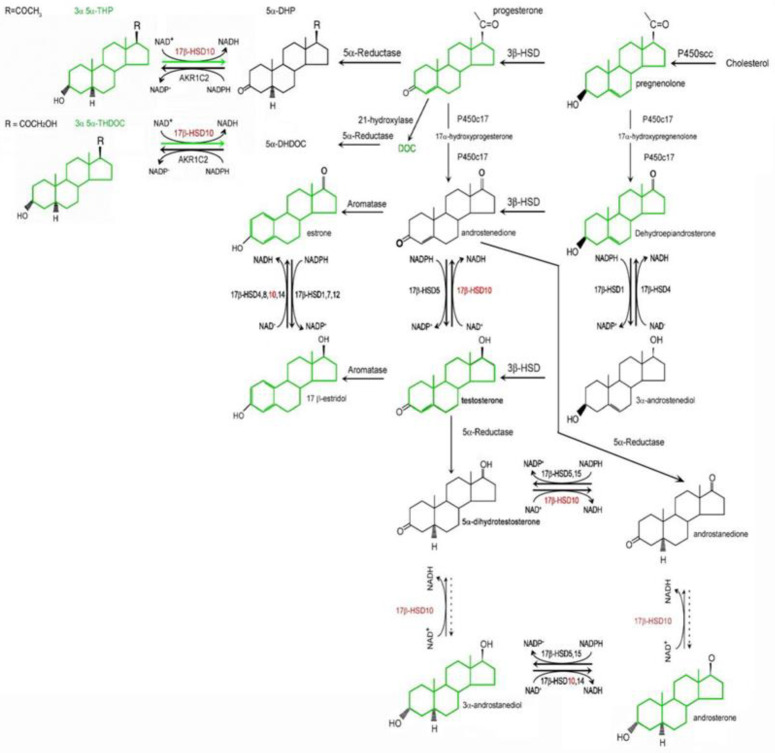
Roles of 17β-hydroxysteroid dehydrogenases in neurosteroid metabolism. Neurosteroids including steroid hormones involved in brain-specific functions are shown in green. Type 10 17β-HSD shown in red, is localized in mitochondria. In contrast, types 2, 3, 6, 7 17β-HSD are in the endoplasmic reticulum while types 1 and 5 17β-HSD in cytosol and type 4 17β-HSD in the peroxisome. Reproduced from Figure 1 of Ref. [51].

**Figure 4:**
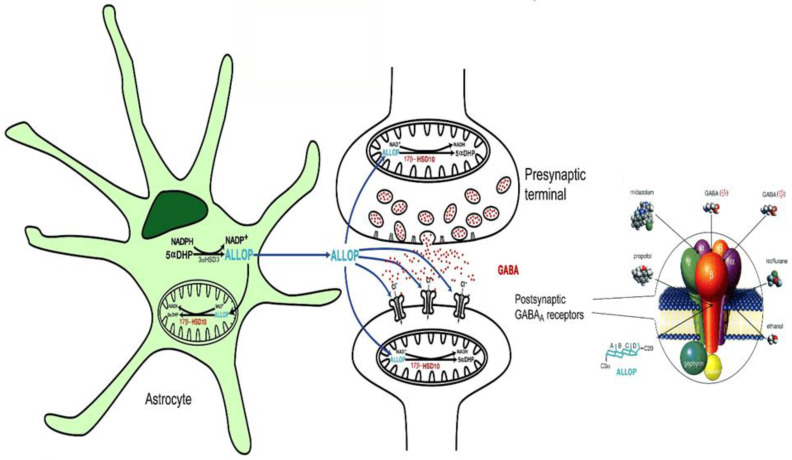
Homeostasis of allopregnanolone (ALLOP) maintained by a dual enzyme molecular switch, consisting of 17β-HSD10 and 3α-HSD3 (AKR1C2). ALLOP is a positive modulator of GABA_A_ receptors that potentiates GABA to increase the opening of Cl^−^ channels. ―• indicates the binding sites of individual modulators on the GABA_A_ receptor. The postsynaptic GABA_A_ receptor was magnified and showed at the right side. Reproduced from Figure 2 of Ref. [51].

Mitochondrial 17β-HSD10 not only catalyzes the oxidation of various acyl-CoA derivatives [33,34,47] (see Figure 2) but also plays an important role in steroid hormone and neurosteroid metabolism [2,3,47,50,51] (see Figure 3). It could modulate neuro-excitability via a *two-enzyme* molecular switch mechanism [51,52], namely 3α-HSD3/AKR1C2 [53] and 17β-HSD10 [52] (see Figure 4). In addition, it could act as cardiolipin phospholipase [54]. However, it needs more in vivo studies to determine [55] whether it plays a role in the metabolism of cardiolipin phospholipid, an important component of the mitochondrial inner membrane [56]. It also acts as a member of the Parkin/PINK1 pathway [57] to control mitochondrial structure and dynamics [58]. Furthermore, it is capable of binding to other proteins to carry out more physiological functions, such as *mt*RNA maturation [59,60,61,62,63,64,65,66,67].

The structure of the *HSD17B10* gene was found to be highly conserved across a broad evolutionary distance [65], as its gene product 17β-HSD10 is vital to life [47,52]. Studies on the properties of 17β-HSD10 are critical to the search for potential treatments of *HSD17B10* gene-related disorders, including infantile neurodegeneration or HSD10 deficiency due to a missense mutation [52,58,66,67,68,69,70,71,72,73,74,75,76,77,78] and intellectual disabilities caused by a silent mutation [79,80] as well as a gene duplication [81]. Elevated levels of 17β-HSD10 were reportedly involved in the pathogenesis of Alzheimer’s disease [11,82,83,84,85,86,87]. Such studies are also important to the elucidation of mechanisms underlying its protective activity in Parkinson’s disease [88,89,90]. The Human Genome Organization (HUGO) announced in 2007 that *HSD17B10* serves as the official symbol of this gene and thus the terminology of the gene product is type 10 17β-hydroxysteroid dehydrogenase [49].

## 4. Enzymatic Activities Regulated by Deacetylation

17β-HSD10 was found to be acetylated at lysine residues (K79, K99 and K105) by the acetyltransferase CREB-binding protein (CBP) and deacetylated by the NAD-dependent deacetylase Sirtuin 3 [91]. Its acetylation regulates its enzymatic activities and the formation of mitochondrial RNase P [91]. The regulation of its intracellular functions affects cell growth and cell resistance in response to stresses [47,88,91].

## 5. Additional Functions Irrelevant to Its Dehydrogenase Catalytic Activities

17β-HSD10 serves as an essential component of the mitochondrial RNase P [50]. It was also found to be a substrate of Parkin, the cytosolic E3 ubiquitin-protein ligase [88], and thus involved in mitochondrial quality control [58,92]. Furthermore, its interaction with cyclophilin D regulates the opening of a mitochondrial permeability transition pore [93].

17β-HSD10 binds to TRMT10C, a methyltransferase, to form a sub-complex catalyzing a conserved m^1^G/A methylation at position 9 of mitochondrial tRNAs [59]. This subcomplex can bind to a single-subunit protein-only RNase P enzyme (PRORP also known as MRPP3) to form mitochondrial RNase P [59,60]. Under these circumstances, 17β-HSD10 is renamed as MRPP2. Here, 17β-HSD10 acts as a platform to play a scaffolding role only as shown in the cryo-EM density map of the *mt*RNase P complex (see Figure 1 of Ref. [55]).

## 6. Re-Discovery of ABAD/ERAB in Mitochondria

As the nucleotide sequence of this gene (AF037438) and its cDNA (AF035555) were deposited into the GenBank in 1997 [1], an article appeared in which a 27 kDa Aβ-binding peptide with 262 amino acid residues was reported to be associated with the endoplasmic reticulum, and thus designated as the endoplasmic reticulum-associated Aβ-binding protein (ERAB) [18] with the attention of media and the focus of some research articles [19,20,21]. As the *HSD17B10* gene product (17β-HSD10/SCHAD) was isolated and demonstrated to be a mitochondrial homo-tetrameric protein, where each subunit consisted of 261 amino acid residues [1,2,3,5,6,7], ERAB was re-designated as Aβ-binding alcohol dehydrogenase (ABAD) based on its so-called *generalized* alcohol dehydrogenase activity *(C2-C10)* [21,22]. At the end of Ref. [21], it was again emphasized that “…*generalized* alcohol dehydrogenase activity, in addition to HADH activity, lead us to propose the new name Aβ-binding protein alcohol dehydrogenase or ABAD to better describe the unusual properties of the enzyme previously referred to as ERAB”. Fortunately, it was found that data underlying such reports are not reproducible (see Figure 5 of Ref. [47]).

The ABAD was first reportedly associated with the *endoplasmic reticulum* (see Figure 2C of Ref. [22]). As a matter of fact, no reliable data to support those ABAD/ERAB reports including Refs. [21,22].

It is still a mystery why the intracellular localization of this protein could be altered from the ER (see Figures 6, 7 and 8a,b of Ref. [21], Figure 4 of Ref. [18] and Figure 2c of Ref. [22]) to mitochondria and then published in the *Science* journal [23] as a new discovery in 2004 by omitting all previous literature already showing the intra-mitochondrial localization of the human [1,2,3,4,5,31,92], mouse [86,87] and rat [32] *HSD17B10* (HADII) gene product—17β-HSD10 or SCHAD.

## 7. An Erroneous Story of Aβ-Binding Alcohol Dehydrogenase

**Figure 5:**
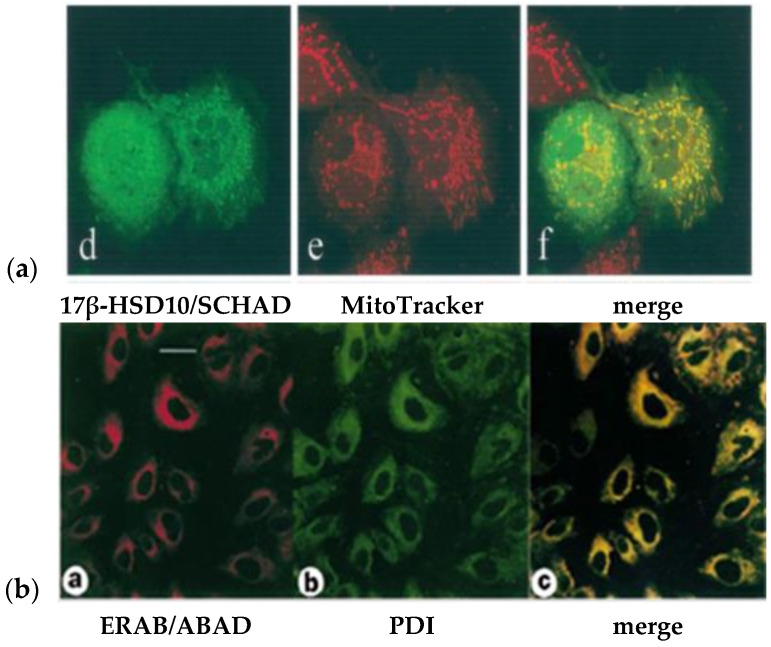
Comparison of the reported intracellular localization data of 17β-HSD10 (**a**) with those of ERAB/ABAD (**b**). In the left column images ((d) green and (a) red) showed the *protein* staining; in the middle column, images ((e) red) and ((b) green) showed the mitochondrial and ER’s staining, respectively. In the right column, image in each low (a) and (b) showed the merge of the left and middle column images (yellow) in the same row. The low (a) is reproduced from Figure 1 of Ref [2]. The low (b) is reproduced from Figure 6 of Ref. [50].

By making a comparison between Figure 5b,c (the reported immune-histological micrographs of ABAD and ERAB, respectively), the following questions emerged. Why are the immune-histological micrographs stained with guinea pig or mouse anti-ABAD [23] completely distinct from those stained with rabbit anti-ERAB/ABAD [18,19,20,21,22]? Since no specific controls [94] were applied in those studies, it is difficult to attribute the action [18,19,20,21,22] to be due to a technical error. More importantly, it was revealed that the subcellular fractionation data for the demonstration of the association of ERAB/HADII with the endoplasmic reticulum [21] were secured by the use of an unreasonable modification of the traditional procedure invented by the Nobel laureate Prof. Christian de Duve (see SM1 and SM2a of Ref. [35]).

The published erroneous data had been formally challenged since 2000 [4] (see SM1 of Ref. [35]), but it appears to be ignored completely by the responsible journal whatsoever. Since ERAB and ABAD both have been recognized as alternate names for the *HSD17B10* gene product, i.e., mitochondrial 17β-HSD10/SCHAD [1,2,3,4,5,31,32,33,34,35,36,37,38,39,40,41,42,43,45,46,47,95], it would be necessary to clarify such erroneous nomenclature and experimental *data* published in prestigious journals without corrigenda [18,19,20,21,22,23,24,25,26,27,28] for scientific research to advance.

### 7.1. Kinetic Constants of ABAD/ERAB Not Based upon Experiments

**Figure 6:**
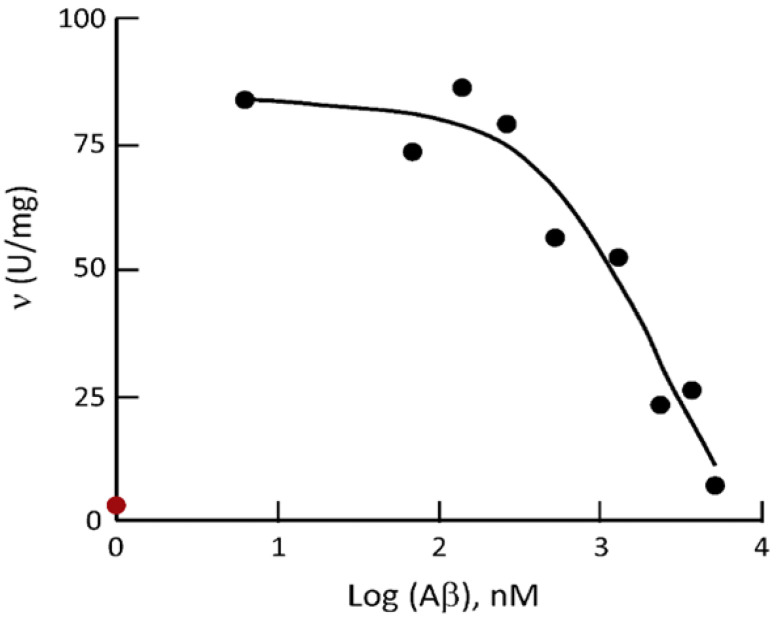
Comparison of the ceiling of HAD activity of human 17β-HSD10 determined by use of the experimental procedure for ABAD/ERAB [21] in the absence of Aβ (red dot) to those reported for ABAD/ERAB under the Aβ_1–40_ inhibition (black dots). Reproduced from Figure 6 of Ref. [47].

Although the intracellular localization of Aβ-binding alcohol dehydrogenase has been transferred from the endoplasmic reticulum [18,19,20,21,22] to mitochondria in 2004 [23] after 17β-HSD10/SCHAD had been isolated from brain mitochondria in 1997 [1], the reported enzymatic properties of ABAD/ERAB [18,19,20,21,22,23,24,25] were always based upon unreliable data (see Figure 6 and Table 1).

Since the enzymatic activity first found in the *HSD17B10* gene product is its 3-hydroxyacyl-CoA dehydrogenase (HAD) activity [1,2,3], human 17β-HSD10 was previously designated as short-chain 3-hydroxyacyl-CoA dehydrogenase (SCHAD) [1,2,3,4,5]. Although the HAD assay had already been well-established [96,97,98], a *novel* experimental procedure for the determination of ERAB/ABAD enzymatic activities was created by authors of Ref. [21] (see Figure 2 of Ref. [47]), and related experimental data were displayed in Table 2 and Figure 3 of Ref. [47]. Unfortunately, such an ABAD/ERAB experimental procedure was found to be nonsense for biomedical research at all (see Figure 2 of Ref. [47]). It was also revealed (see Figures 4 and 5 of Ref. [47]) that the reported kinetic constants (see the right column of Table 2 reproduced from Ref. [47]) are ***not*** based upon real assays. In other words, data are *not* secured by experimental studies, or *no* experiments had been performed according to the experimental conditions described in the same scientific report namely Ref. [21].

In that controversial publication, the reaction was reportedly catalyzed by ERAB (333 ng/mL). If the *reported kinetic constants* of ERAB/ABAD [21] are taken for granted, the enzymatic reaction would already be completed before the first data point was observed at 5 min. Although the change of A_340_ with time was observed at 5 min for the oxidation of 17β-estradiol catalyzed by 17β-HSD10/SCHAD (see Figure 3a of Ref. [35]), it is certainly ***not*** so in the reduction of S-acetoacetyl-CoA by NADH, because the V_max_ of 17β-HSD10/SCHAD for catalyzing this hydrogenation reaction of S-acetoacetyl-CoA is much greater than that for its catalyzing of 17β-estradiol oxidation by about thirty-three hundred folds [3,4,5].

Although the v was proportional to [S] when [S-acetoacetyl-CoA] < 0.1 mM, it was certain that the v would have little increase with the increased [S-acetoacetyl-CoA] if it is greater than 0.1 mM because of the limitation of [NADH] in the assay system (see Figure 4 of Ref. [35]).

Although the assay designers of Ref. [13] appeared to disregard the basic enzymology concept of the *initial velocity* [99], a much more significant problem here is the *inconsistency* between published *data* and the reported *experimental procedures*, no matter whether the reported experimental procedures of the *JBC* article [21] are scientifically rational or not.

A comparison of the actual kinetic constants of the *HSD17B10* gene product, 17β-HSD10, to those reported for ERAB/ABAD [21,22] was shown in Table 2. Although the ERAB problem had first been exposed two decades ago [4] (see SM1 of Ref. [35]), it was continuously covered by the related journals not making the necessary corrections. In this way, serious problems become ‘an old issue’ (see SM2 of Ref. [35]). The related editor took the so-called ‘old issue’ as an excuse for their omission, although an associate editor had previously promised that the editorial board would deal with the issue after the celebration of the 100th anniversary of the Journal.

### 7.2. ‘Competitive Inhibition’ of Aβ Defined by a Single Concentration of Substrate

It was reported [52] that a 17β-HSD10 mutant behaves as an allosteric enzyme with a Hill coefficient of about 1.3. Further investigation of the inhibition of the *HSD17B10* gene product, 17β-HSD10, by Aβ is underway. It was reported to be a *one-site competitive inhibition* [21], and an equation for calculating the K_i_ value was recently released (see SM2 of Ref. [35]). However, it was found that only a single concentration of substrate and a single concentration of coenzyme, i.e., 0.18 mM S-acetoacetyl-CoA and 0.1 mM NADH as described in the legend for Figure 5 of Ref. [21], had been employed for the inhibition study on an ordered Bi-Bi reaction [99]. As a result, it is hard to know what kind of inhibition it could be (see Figure 5A of Ref. [35]). There is no reason to believe what was claimed by the authors of Ref. [21], a so-called ‘one-site model for competitive inhibition’, until more studies are completed as those recently reported for the inhibitor, benzothiazolyl ureas [100].

**Figure 7:**
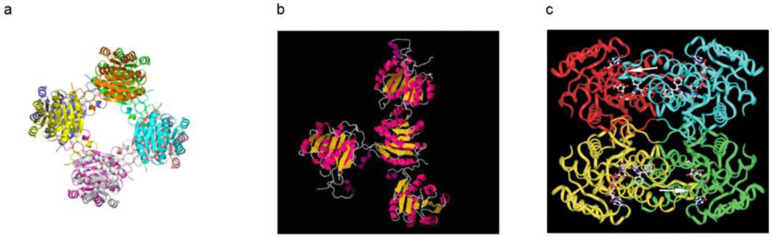
A comparison between three-dimensional structures of Aβ bound ABAD [23] (**a**,**b**) and that of 17β-HSD10/ABAD [50] (**c**).

The X-ray diffraction study on rat ABAD (17β-HSD10) revealed its tetramer structure [30]. Information about the three-dimensional structure of the human protein is available in 2004 [23] (PDB:1U7T, see Figure 7c). However, it is uncertain whether binding of Aβ could lead to such radical changes of the 3D structure of 17β-HSD10, e.g., from what is seen in Figure 7c to that in Figure 7b displayed in Ref. [23] (PDB:1S08). A dramatic increase of the protein surface would need a supply of much energy and such a high energy status is very unstable. Since no electron density of Aβ was observed and described in that *Science* article [23], and since there is a clear difference between the reported 3D structures of Aβ-bound ERAB/ABAD if making a comparison between Figure 7a,b, the reliability of the reported Aβ-bound ERAB/ABAD structure [23] is questionable. In addition, the question of whether there is a large solvent channel in the center of ABAD [23] (see Figure 7a) also needs to be clarified by further studies.

As the pioneers in the research field of the human *HSD17B10* gene and its product 17β-HSD10 [1,2,3,4,5,31,32,33,34], we eventually received an official reply from the *JBC* editorial board after about two decades (see SM2 of Ref. [35]), in which the so-called *one-site competitive inhibition* equation was shown below.

(v/V_max_) = S/[K_s_ (1 + (I/K_i_)) = S] or some derivative (1)

Surprisingly, it was found in this ‘rate equation’ that its *left* side, v/V_max_, was not equal to the *right* side. This rate equation cannot illustrate competitive, noncompetitive or uncompetitive inhibition (see SM2 of Ref. [35]). If careful corrections are made, it might be applicable to a competitive inhibition of the reaction catalyzed by a monomeric enzyme [99], but it is still useless for studies on the inhibition of 17β-HSD10 by Aβ (see Figure 5 of Ref. [21]) since substituting Log[I] for I as proposed by some *JBC* editorial board member is not warranted. 

It was further stated in the questionable ABAD/ERAB report [21] that the data analysis was “using a one-site model by the method of Klotz and Hunston”. Since that literature’s title was found to be ‘Mathematical models for ligand-receptor binding. Real sites, Ghost sites’ [101], and the ABAD/ERAB researchers [18,19,20,21,22,23,24,25] should provide answers to the following questions:

(1) Whether the so-called ‘one-site’ model [21] refers to one of the real sites or ghost sites?
(2) Is it possible that the use of the Klotz and Hunston method [102] could simplify the related ‘competitive’ inhibition study [21] to the use of a single substrate concentration only?

### 7.3. Non-Reproducibility of Reported ABAD Assays

**Figure 8:**
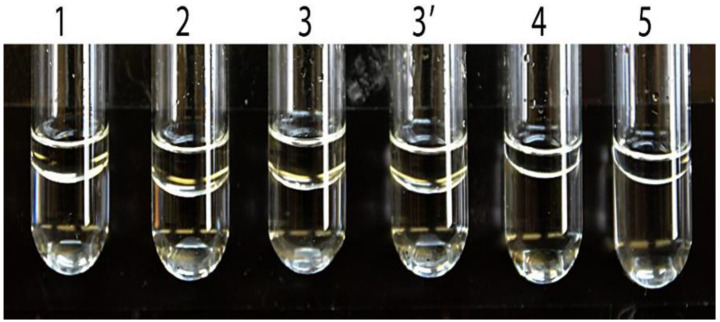
ABAD assay mixture prepared according to the published Experimental Procedure (see SM1 of [13]) with (−)-2-octanol, 160 mM (tube 1), 210 mM (tube 2), 210 mM and 1% Me_2_SO (tube 3), 210 mM mixed first with 1% Me_2_SO (tube 3′), or with 84 mM (+)-2-octanol ((tube 4), and 85 mM (±)-2-octanol (tube 5), respectively. All tubes were vortexed at high speed for 3 min and then incubated at room temperature (25 °C) for 72 h. The interface between two layers in the assay mixture is noticeable. Reproduced from Figure 5 of Ref. [47].

It is well known that alcohols with an alkyl >6 carbon have poor solubilities in water. (−)-2-Octanol, (+)-2-octanol and (±)-2-octanol are oils at 25 °C. Since the solubility of (−)-2-octanol and (+)-2-octanol are only 6 mM and 8.5 mM, respectively [103], the solubility of racemic (±)-2-octanol could not be greater than 15 mM. As shown in Figure 8, it is not possible to determine ABAD activity towards 2-octanols spectrophotometrically by following the published Experimental Procedures [21] (see SM3b and 3c of Ref. [35]), especially because the reported K_m_ values of ABAD/ERAB for (−)-2-octanol, (+)-2-octanol and (±)-2-octanol were as high as 43, 86 and 87 mM, respectively.

An interface between the layers does not disappear after the addition of 1% Me_2_SO to the assay system and being vortexed at high speed for 3 min and then incubated at room temperature (25 °C) for 72 h (see Tubes 3 and 3′ in Figure 8). The results are just opposite to the prediction by some members of the *JBC* editorial board who insisted on there being nothing wrong in those *JBC* articles [21,22]. Experimental details have been described in the SM3c of Ref. [35] for researchers who are interested in confirming the findings (see Figure 8). As a result, no one knows how the previously reported data and the published Figures 2B and 5B of Ref. [21] could have been obtained by assays performed under the reported experimental condition (see SM3c of Ref. [35]). Obviously, no reliable data support the conclusion that the *HSD17B10* gene product exhibits *generalized* alcohol dehydrogenase activity (C2-C10) (see SM3b of Ref. [35]), which underlies the conversion of ABAD from ERAB [15,16,17,18,19,20,21,22,23,24,25,26,27]. This was the subject of a *JBC* report [22] and thereafter dozens of ABAD reports have appeared in various journals until the present time [26,27,28,29]. The term ABAD or ERAB, which probably originated from unreliable data, whenever used for the *HSD17B10* gene product should be replaced by 17β-HSD10 without exception.

### 7.4. Importance of ABAD for Brain Cells’ Resistance to Oxidative and Nutritional Stress?

The ABAD/ERAB was reportedly contributing to the protective response to metabolic stress, especially in the setting of ischemia, because it could catalyze the oxidation of the ketone body D-3-hydroxybutyrate with K_m_ of 4.5 mM and V_max_ of 4 nmol/min/mg protein [21,22] (Only prokaryote**s**’ 3-hydroxyacyl-CoA dehydrogenase can catalyze the dehydration of both L- and D-isomers [104]). However, ABAD/ERAB was reportedly associated with the endoplasmic reticulum in the same *JBC* report (see Figure 2C of Ref. [22]). Most of the cell’s NAD^+^ is enclosed in mitochondria. It is hard to know whether such an ‘*ER-associated NAD^+^-dependent dehydrogenase*’ namely ERAB/ABAD could, indeed, play a physiological role in the ketone body metabolism. Furthermore, biochemists are generally aware that mitochondrial β-hydroxybutyrate dehydrogenase, β-HBD [105] rather than ABAD [21,22] or 17β-HSD10/HADII [31,32,33,34,35,36,37,38] is involved in ketone body metabolism (see Figure 2). Although data about D-3-hydroxybutyrate supporting the growth of COS cells were provided previously (see Figure 5 of ref. [22]), it could not serve as valid evidence for ABAD’s involvement in ketone body metabolism because β-HBD had not yet been knocked out from the COS cell line used by Yan et al. [22].

It had already been reported [1] that SCHAD/17β-HSD10 metabolizes only L-3-hydroxyacyl-CoA but not the D-isomer. Obviously, the report that ABAD catalyzes the oxidation of ketone bodies and, therefore, contributes to the protective response to metabolic stress, especially in the setting of ischemia [21,22] was also based upon *non-reproducible* data. Some possible mechanisms of 17β-HSD10, other than the oxidation of the ketone body, to play a role in the regulation of cell growth and cell resistance under oxidative and starvation stresses were reported recently [91]. After 17β-HSD10 proved to be an important mitochondrial enzyme (see Figure 2 and Figure 4), people are asking whether mitochondria are dysfunctioning in Alzheimer’s disease [47,58,106] and how we could find a way to deal with progressive neurodegeneration [58,107,108,109,110].

## 8. *HSD17B10* Gene-Related Disorders (OMIM:#300438)

The expression of the *HSD17B10* gene to generate an appropriate amount of 17β-HSD10 in different tissue cells, especially in different brain regions is critical to human health [111]. Either a mutation on this gene resulting in 17β-HSD10 mutants or the abnormal expression of this gene could cause human diseases such as infantile neurodegeneration [52] and senile neurodegeneration [112,113].

### 8.1. About Half Cases of HSD10 Deficiency Resulting from a p.R130C Mutation

About half of the cases of this disease are due to a missense C > T in exon 4 of the *HSD17B10* gene, since the +2259 nucleotide from the ATG of this gene is >90% methylated in human X chromosome [114,115,116]. The 5-methylcytosine is prone to conversion to thymine by deamination. The substitution of arginine for cysteine eliminates several hydrogen bonds and reduces the *van der Waals* interaction between HSD10 subunits. HSD10 mutant could cause human diseases such as infantile neurodegeneration [52] and senile neurodegeneration [47,51,88]. Aβ levels in the cerebrospinal fluid of HSD10 deficiency patients are undetectable [117]. It indicates that the pathogenesis is most likely *not* due to the mediation of Aβ neurotoxicity by 17β-HSD10. 

### 8.2. Few Female Cases of HSD10 Disease Because of X-Inactivation

**Figure 9:**
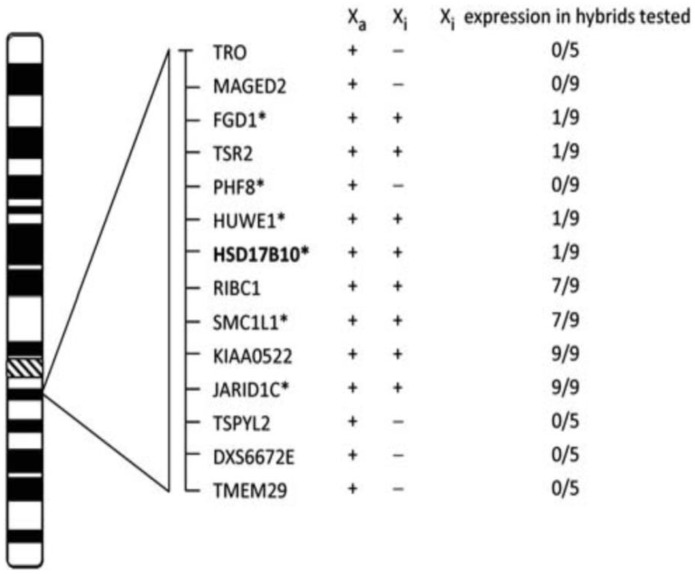
Expression of transcripts of *HSD17B10* and surrounding genes from inactive X (Xi) hybrids. Samples scored as positive are expressed at least 10% of the Xa levels, and their number is shown as the numerator. The total number of hybrids tested is shown as the denominator. Gens with mutation(s) or copy number variation (CNV) causing mental retardation are marked with an asterisk. Reproduced from Figure 1 of Ref. [118].

People who suffer from HSD10 disease are mostly male (see Figure 5 of Ref. [75]). Female cases with HSD10 deficiency were at least ten times less than male patients [72], because the female possesses two X-chromosomes. The random X-inactivation skewing mechanism [114,115] may randomly suppress the expression of the HSD10 mutant (see Figure 9). Clinical manifestations of some female HSD10 disease patients are also milder [52]. However, sons of the female carriers of *HSD17B10* mutations are at high risk of suffering from this inherited metabolic disease.

### 8.3. Elevated Levels of 17β-HSD10 in Brain Cells of AD Patients or down Syndrome Patients with AD Pathology

**Figure 10:**
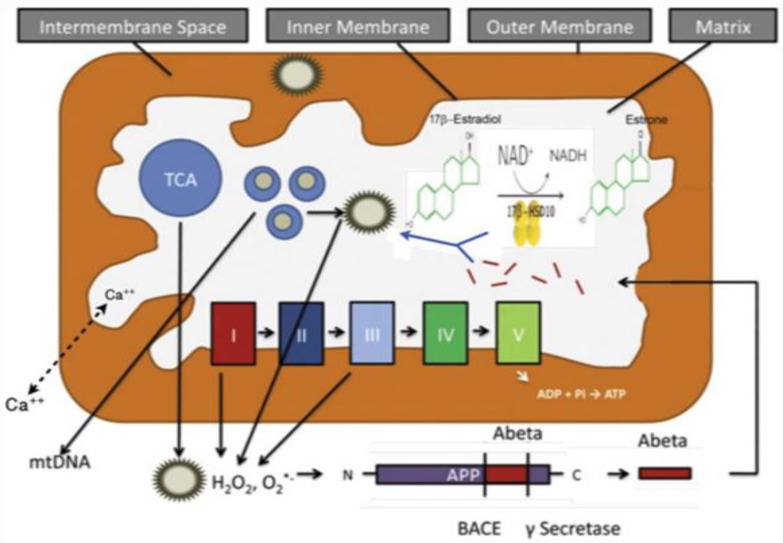
Oxidative inactivation of neuroprotective neurosteroid 17β-estradiol by 17β-HSD10 in brain mitochondria. The I, II, III, IV and IV represent the complexes consisting of the electron-transport chain for the oxidative phosphorylation to generate ATP. Updated from Figure 1 of Ref. [58].

Most people with Down’s syndrome develop AD-like dementia by the fifth to sixth decade of life, a much younger age than is typically seen in sporadic, late-onset AD [119]. The *HSD17B10* gene product, 17β-HSD10, catalyzes the oxidative inactivation of 17β-estradiol to estrone [2]. Elevated levels of the *HSD17B10* gene product, 17β-HSD10, found in brain cells of Down’s syndrome patients with AD pathology (see Figure 1 of Ref. [120]), would accelerate the oxidative inactivation of the protective neurosteroid, 17β-estradiol (see Figure 10) and thus be harmful to the function and structure of brain mitochondria.

### 8.4. HSD10 Inhibitors as Potential Candidates for Treatment of Senile Neurodegeneration

There is no effective way to deal with increasing cases of the related neurodegeneration. Since 17β-HSD10 is involved in brain cells’ metabolism including neurosteroid metabolism and elevated levels of HSD10 were found in the brains of AD patients and AD mice models, inhibitors of this vital mitochondrial enzyme may be useful to alleviate the progress of neurodegeneration [106]. Whether such an inhibitor could effectively prevent mitochondrial dysfunction is considered an essential criteria for further investigation [107,108,109,110].

## 9. Perspective

Research in the field of the *HSD17B10* gene product has been severely interfered with by ABAD/ERAB publications shown in prestigious scientific journals for two decades. No one should have the right to cover errors in their publications without corrigenda to prevent their readers from being misled. Nevertheless, consistent efforts of honest scientists in this field have resulted in significant advances such that ‘17beta-hydroxysteroid dehydrogenase X’ became the title of OMIM300256 and *HSD17B10* has been approved by the human genome nomenclature committee (HGNC) as the gene symbol. It would encourage people to determine why elevated levels of 17β-HSD10/SCHAD, besides Aβ and phosphorylated Tau, are present in the brains of AD and some Down’s syndrome patients with AD pathology as well as mouse AD models. Such efforts may open new approaches to the finding of treatments for neurodegeneration seen in AD and Parkinson’s disease. 

## Figures and Tables

**Table 1:** Alternative designations of human *HSD17B10* gene product ^‡^.

Year	Accession Number		Name	Acronym	Comments	Ref.
	cDNA	Gene				
1997–1998	**AF035555 AF037438** **[11/21/97] [12/9/97]** **Deposited into the Genbank respectively**		Short-chain 3-hydroxyacyl CoA dehydrogenase	SCHAD	**MW = 108 kDa, composed of 1044 residues. Homotetrameric enzyme exhibits HAD activity and proposed to reside in mitochondria**	[1]
	U96132 n/a		Endoplasmic reticulum-associated Aβ-binding protein	ERAB	MW = 27 kDa, **composed of 262 amino acid residues and associated with endoplasmic reticulum (ER)**	[18]
1999			Novel 17β-Hydroxysteroid dehydrogenase	Novel 17β-HSD	**Mitochondrial, multifunctional protein** inactivates 17β-estradiol to estrone	**[2]**
			Amyloid β-peptide binding alcohol dehydrogenase	ABAD	Substitution for ERAB but it still associated with ER and **to further convey incongruous data of *generalized* alcohol dehydrogenase (C2-C10) activities**	[21]
2000			2-methyl-3-hydroxyacyl-CoA dehydrogenase	MHBD	Appropriate for the isoleucine metabolism	**[8]**
2001	**OMIM300256:** **17beta-Hydroxy-** **steroid dehydrogenase X**		**Type 10 17β-Hydroxy-steroid dehydrogenase**	**17β-HSD10**	**Identification of its N-terminal mitochondrial targeting signal**	**[3]**
2004			Amyloid β-peptide binding alcohol dehydrogenase	ABAD	Claimed to change the ER-associated ABAD to be a mitochondrial ABAD without any citations of 17β-HSD10/SCHAD literature	[23]
2007	NM_004493, Gene symbol: *HSD17B10* *		3-Hydroxyacyl-CoA dehydrogenase type 2	HADH2	A silent mutation was found in MRXS10 ** patients	[80]
2008			Mitochondrial RNase P protein 2	MRPP2	In RNA-free RNase P complex	[59]
2013			Short-chain dehydrogenase/reductase 5C1	SDR5C1	From a short-chain de-hydrogenase/reductase (SDR) evolution tree	[37]

^‡^ Updated from Table 1 of Ref. [35]; * This official gene name *HSD17B10* substituted for the *HADH2* [49]. ** Mental retardation X-linked syndromic 10 [80].

**Table 2:** Comparison of catalytic constants of 17β-HSD10 and ABAD/ERAB.

Substrate	17β-HSD10 *			ABAD/ERAB ^¶^		
	*k*_cat_ (s ^−1^)	K_m_ _(µM)_	*k*_cat_/K_m_ (s^−1^/_µM_)	*k*_cat_ (S^−1^)	K_m_ _(µM)_	*k*_cat_/K_m_ (s^−1^/_µM_)
***Reduction by NADH:***						
S-Acetoacetyl-CoA	37	89	0.42	190	68	2.8
***Oxidation by NAD^+^:***						
D-β-hydroxy-butyrate	Not detectable	―		0.40 × 10^−6^	4500	8.9 × 10^−12^
Ethanol	Not detectable	―		0.82 × 10^−6^	1.21 × 10^6^	6.8 × 10^−13^
(−)-2-octanol ^‡^	ND	―		1.3 × 10^−3^	43 × 10^3^	3.0 × 10^−8^
17β-Estradiol	11 × 10^−3^	43	2.6 × 10^−4^	100	14	7.1 × 10^−1^

* Data from Refs. [1,3,35]. ^¶^ Data shown in Refs. [21,22] were recently revealed *not* secured obtainable from reported experimental studies. ^‡^ Not measurable by use of the experimental procedure reported in Refs. [21,22], because the actual saturated concentration of (−)-2-octanol is 8.6 mM only. ND, Not determined spectrophotometrically.

## Abbreviations

ABAD	Aβ-binding protein alcohol dehydrogenase
AD	Alzheimer’s disease
ERAB	Endoplasmic Reticulum-associated Aβ-binding protein
HADH	L-3-hydroxyacyl-CoA dehydrogenase
HADII	type II 3-hydroxyacyl-CoA dehydrogenase
HSD	hydroxysteroid dehydrogenase
17β-HSD10	17β-hydroxysteroid dehydrogenase type 10
Me_2_SO	dimethyl sulphoxide
*mt*	mitochondrial
MRPP	mitochondrial ribonuclease P protein
MRXS10	mental retardation
X-linked	syndromic 10
PD	Parkinson’s disease
PDI	protein disulfide isomerase (an ER marker)
PRORP	protein only RNase P
OMIM	Online Mendelian Inheritance in Man
SCHAD	short-chain 3-hydroxyacyl-CoA dehydrogenase
SM	Supplementary materials
TRMT10C	methyltransferase 10C
VDAC	anti-voltage-dependent anion channel (a mitochondrial marker)
